# Web-Based Benefit-Finding Writing for Adults with Type 1 or Type 2 Diabetes: Preliminary Randomized Controlled Trial

**DOI:** 10.2196/13857

**Published:** 2019-06-27

**Authors:** Joanna Crawford, Kay Wilhelm, Judy Proudfoot

**Affiliations:** 1 Black Dog Institute Sydney Australia; 2 School of Psychiatry University of New South Wales Sydney Australia; 3 Consultation Liaison Psychiatry St. Vincent’s Health Australia Sydney Australia

**Keywords:** diabetes, adult, distress, benefit-finding, depression, anxiety, emotions, internet, writing, surveys and questionnaires, treatment outcome

## Abstract

**Background:**

The high prevalence of diabetes distress and subclinical depression in adults with type 1 and type 2 diabetes mellitus (T1DM and T2DM, respectively) indicates the need for low-intensity self-help interventions that can be used in a stepped care approach to address some of their psychological needs. However, people with diabetes can be reluctant to engage in mental health care. Benefit-finding writing (BFW) is a brief intervention that involves writing about any positive thoughts and feelings concerning a stressful experience such as an illness, avoiding potential mental health stigma. It has been associated with increases in positive affect and positive growth and has demonstrated promising results in trials in other clinical populations. However, BFW has not been examined in people with diabetes.

**Objective:**

This study aimed to evaluate the efficacy of a Web-based BFW intervention for reducing diabetes distress and increasing benefit finding in diabetic adults with T1DM or T2DM compared to a control writing condition.

**Methods:**

Adults with T1DM or T2DM and diabetes distress were recruited online through the open access Writing for Health program. After completing baseline questionnaires, they were randomly allocated to receive online BFW or an active control condition of online writing about the use of time (CW). Both groups completed 15-minute online writing sessions, once per day, for 3 consecutive days. Online measures were administered at baseline, 1 month, and 3 months postintervention. Participants were also asked to rate their current mood immediately prior to and following each writing session.

**Results:**

Seventy-two adults with T1DM or T2DM were recruited and randomly allocated to receive BFW (n=24) or CW (n=48). Participants adhered to the BFW regimen. Greater increases in positive affect immediately postwriting were found in the BFW group than in the CW group. However, there were no significant group-by-time interactions (indicating intervention effects) for benefit finding or diabetes distress at either the 1-month or 3-month follow-up. Both the BFW and CW groups demonstrated small, significant decreases in diabetes distress over time.

**Conclusions:**

BFW was well tolerated by adults with diabetes in this study but did not demonstrate efficacy in improving diabetes distress or benefit finding compared to an active control writing condition. However, due to recruitment difficulties, the study was underpowered and the sample was skewed to individuals with minimal diabetes distress and none to minimal depression and anxiety at baseline. Future research should continue to investigate the efficacy of variants of therapeutic writing for adults with T1DM or T2DM, using larger samples of participants with elevated diabetes distress.

**Trial Registration:**

Australiand New Zealand Clinical Trials Registry ACTRN12615000241538; https://www.anzctr.org.au/Trial/Registration/TrialReview.aspx?id=368146

## Introduction

### Background

Diabetes mellitus is group of disorders posing a global public health challenge. In 2018, the International Diabetes Federation estimated that the global prevalence of diabetes in adults in 2017 was 8.4% (451 million adults) [1]. Psychological comorbidity is common and burdensome in people with type 1 or type 2 diabetes mellitus (T1DM or T2DM, respectively) [2-4], with many experiencing negative thoughts and emotions toward diabetes and its treatment [4,5]. Diabetes distress is a construct correlated with, but distinct from, depression in people with diabetes and includes distress associated with the treatment regimen, eating, hypoglycemia, complications, interpersonal relationships, and health care professionals [5,6]. Approximately 70% of people with T2DM display high levels of diabetes-related distress without meeting the criteria for major depressive disorder (MDD) [7,8]. Diabetes distress is associated with poor glycemic control and acts as a unique contributor to poor diabetes self-care [9,10]. Several studies have found that diabetes distress mediates the association between depressive symptoms and hemoglobin A_1c_ (HbA_1c_) [11,12]. Further, diabetes distress is also a risk factor for depression [13].

Similarly, in people with diabetes, anxiety and depression are associated with poorer diabetes self-management [14-17] and increased disease severity and complications [16-19]. Of note, subthreshold depressive symptoms (which fall short of the full diagnostic criteria for MDD or dysthymia) are more common in people with diabetes than MDD, but are less likely to be diagnosed than MDD [20]. Approximately half of the people with T2DM will experience at least one episode of subthreshold depression over 5 years [19]. Subthreshold depressive symptoms in people with diabetes are associated with poorer quality of life [21,22] and poorer diabetes self-care [15]. These findings have increased attention to addressing diabetes distress and mild depressive symptoms in patients with diabetes and may provide an avenue for preventing MDD [13,21,23] and improving diabetes self-management [11,12,24].

The international guidelines for diabetes management now recognize the importance of psychological care [24]. Screening for both diabetes distress and depressive symptoms in people with diabetes, followed by appropriate interventions, has been recommended [21,25]. A stepped-care approach has been suggested, with mild distress or subthreshold depression managed within primary care, utilizing evidence-based self-help interventions [26,27]. Web-based interventions, predominantly clinician-assisted interventions, are effective for reducing comorbid depression in people with diabetes [28]. However, many interventions for diabetes distress or depression rely upon face-to-face group sessions [29], numerous telephone calls [30], or clinician support [28]. Overall, there is growing recognition of the need for low-intensity interventions to address diabetes distress or subthreshold depression, which are cost-effective and easily disseminated to large numbers of patients.

However, many people with diabetes are reluctant to seek mental health care [31]. This may be due to the stigma of mental ill health and the belief that distress associated with diabetes is normal and should not be pathologized [32]. Hence, interventions that do not explicitly refer to “depression” or “anxiety” may appeal more to some people with diabetes [32].

Therapeutic writing is a brief intervention that aims to improve physical or mental health [33]. The most common form of therapeutic writing is expressive writing (EW), in which thoughts and feelings regarding a stressful event are disclosed in writing, typically for 15-20 minutes for 3-4 days within a short period of time [34]. EW has been examined in over 250 studies investigating its effects on physical or mental health in a wide range of populations, including healthy participants, people with psychological problems, and people with chronic health conditions [33,35-37]. However, there are limitations to EW. Results of EW studies are quite variable, and the effect sizes are often small [33,35]. Further, EW often involves an immediate increase in distress [33], even when followed by longer-term benefits [38], which limits its suitability for wide dissemination without therapist support.

Indeed, trials of EW in people with diabetes have yielded mixed results. One study, for example, found fewer depressive symptoms at follow-up [39], while another found reduced stress but no effect on HbA_1c_ levels, diabetes self-care, or diabetes distress [40]. A later trial found that EW was associated with a *worsening* in depressive symptoms, with no change in diabetes distress [41]. Of note, in the latter study, the EW task involved writing about any stressful experience over the past month rather than a diabetes-specific task.

Such findings have led researchers to investigate other variations of therapeutic writing, to maximize benefits and increase positive affect (and reduce distress) during the intervention. By modifying writing instructions, researchers have sought to increase the likelihood that participants engage in desired cognitive processes, with the aim of increasing the benefits gained from the writing task [42,43]. The integration of positive psychology into therapeutic writing is one such modification [44].

Benefit-finding writing (BFW) involves writing about any *positive* thoughts and feelings about a stressful experience such as an illness. Until recently, research has largely overlooked the utility of positively focused writing following stressful events or illness. However, there is emerging evidence that the experience of a medical illness often has sequelae that patients view as positive or beneficial [45]. Benefit finding is defined as “identifying positive life changes resulting from adversity and negative life stressors, including illness” [46]. It is correlated with posttraumatic growth [47], which has recently been found to be associated with greater positive affect and less negative affect in the daily lives of people with some chronic health conditions [48]. Benefit finding has been associated with increased psychosocial well-being and decreased depression in a range of clinical populations [46,49]. Benefit finding in diabetes has been associated with lower symptoms of depression, increased adherence to diabetes self-care, and greater perceived coping effectiveness [50]. Therefore, interventions to increase benefit finding in people with diabetes could be useful [50].

Trials of BFW in both nonclinical populations [43,51-54] and clinical populations [55-58] have demonstrated promising results. Compared to EW, BFW has been found to result in less distress and increased positive affect immediately postwriting [51,59]. Results from pilot trials in clinical populations suggest that BFW has benefits with regard to the symptoms of depression [56], anxiety [55], fatigue [58], and the number of cancer-related medical appointments [57]. In addition, positive affect journaling has recently been found to reduce symptoms of depression and anxiety in general medical patients with elevated anxiety [44]. Thus, the limited research on BFW to date suggests that it may have the same longer-term health benefits as EW, but with the added advantage of immediate increases in *positive* affect. However, BFW has not been examined in people with diabetes.

To our knowledge, this is the first study of BFW in people in diabetes. We aimed to examine BFW in adults with T1DM or T2DM who were experiencing diabetes distress, but were not experiencing a mood or anxiety disorder. This is because people with diabetes who meet the criteria for MDD or an anxiety disorder are likely to require more intensive treatment, with several evidence-based interventions available [28]. Our study included participants reporting mild or minimal symptoms of depression or anxiety, in addition to any degree of diabetes distress.

### Objectives

The primary aim of this randomized controlled trial (RCT) was to evaluate the efficacy of a Web-based BFW intervention for adults with T1DM or T2DM (compared to a CW condition) for reducing diabetes distress and increasing benefit finding in diabetes. The secondary outcomes examined were self-rated depression and anxiety symptoms, diabetes self-care, health, and health care utilization. It was hypothesized that participants randomized to the BFW condition would demonstrate significant decreases in diabetes distress and symptoms of depression and anxiety, and report reduced visits to health care professionals compared to those in the control group. It was also hypothesized that participants randomized to the BFW condition would experience significant increases in benefit finding in relation to diabetes and improvements in diabetes self-care and perceived health, relative to those in the control group, and that these effects would be evident at 1-month and 3-month follow-up assessments.

This study also aimed to conduct manipulation checks of the BFW intervention instructions by investigating immediate emotional responses to the writing tasks and their linguistic content. It was hypothesized that, compared to those in the CW group, participants in the BFW group would (1) have significantly greater increases in positive affect immediately after writing but not significantly different changes in negative affect; (2) use a significantly higher proportion of positive emotion words, negative emotion words, and cognitive processing words, in line with previous research [51]; and (3) rate their responses as significantly more personal and more meaningful, but not more distressing.

Finally, as people with diabetes can be reluctant to engage in psychological care [31], this study aimed to examine the feasibility of internet-based BFW by adults with T1DM or T2DM. Feasibility was assessed by adherence and acceptability of the intervention.

## Methods

### Design

The design consisted of a CONSORT-EHEALTH [60]–compliant, 2 (conditions) x 3 (time) RCT (Multimedia Appendix 1). The protocol for the trial has been published [61]. Participants were randomized to either internet-based BFW or internet-based CW (use-of-time writing). Both conditions involved an intervention of 3 days of online writing using the *Writing for Health* website (see below). Outcome measures were administered at three time points for both groups: baseline, 1 month, and 3 months postintervention. Self-rated current mood was also assessed immediately prior to and following each writing session. An online feedback questionnaire was administered postintervention.

### Participants and Recruitment

Ethics approval was obtained from the Human Research Ethics Committee at St. Vincent’s Hospital, Sydney, Australia (HREC/13/SVH/379). All participants provided informed consent before engaging in any research-related activity. This study was prospectively registered with the Australia and New Zealand Clinical Trials Registry (ACTRN12615000241538).

The study was advertised from February 2015 to November 2016 on the websites, social media, and publications of Australian diabetes-related organizations and advertisements in waiting rooms of diabetes services and general practitioners throughout Sydney, Australia. Due to slow recruitment, the study was also advertised in Diabetes Australia’s printed Circle Magazine, which was mailed to 140,000 members. Finally, letters were mailed to 500 adult members of the National Diabetes Services Scheme in Australia, informing them of the study. In all advertising and recruitment materials, adults with T1DM or T2DM were directed to the *Writing for Health* website to find out more about the study.

Eligibility and exclusion criteria for adults with T1DM or T2DM living in Australia are shown in Textbox 1. The exclusion threshold was set at 8 or above on both the Generalized Anxiety Disorder-7 items (GAD-7) scale or the Patient Health Questionnaire-9 items (PHQ-9) scale, as sensitivity and specificity values have been found to be acceptable for a cutoff of 8 on the GAD-7 scale for identifying generalized anxiety disorder [62] and the PHQ-9 scale for identifying MDD [63]. Participants were not provided any compensation for taking part in the study.

**Eligibility and exclusion criteria.**

Eligibility criteria:

- Consent to participate
- Age ≥ 18 years
- Living in Australia
- Type 1 or type 2 diabetes, self-reported as diagnosed by a general practitioner or endocrinologist
- Email address and access to the internet
- Ability to read and write in English with ease

Exclusion criteria:

- Patient Health Questionnaire-9 score >8 or Generalized Anxiety Disorder-7 score >8
- Current suicidal thoughts, as indicated by a response of >1 to item 9 on the Patient Health Questionnaire-9 scale
- Self-reported diagnosis of schizophrenia, bipolar disorder, or a psychotic disorder
- Self-reported diagnosis of dementia or another cognitive disorder
- Engagement in current psychological therapy

### Procedure

**Figure 1:**
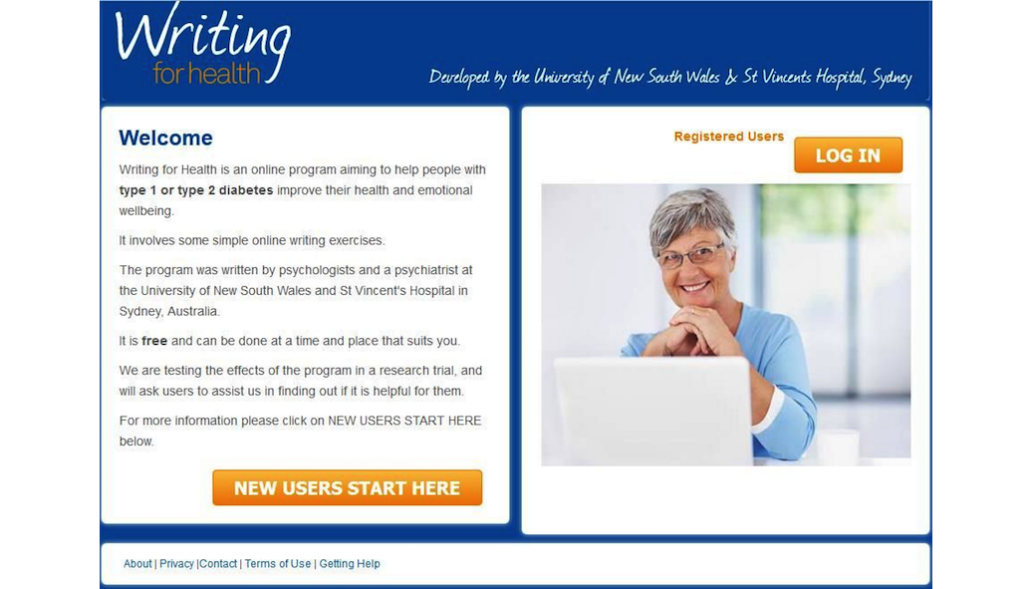
Homepage of Writing for Health.

Potential participants applied for the study via the open access *Writing for Health* website (Figure 1). All stages of this study were conducted online through *Writing for Health*, including information about the study, consent to participate, screening questionnaires with automated feedback, participant registration, randomization to one of two conditions, the writing interventions, feedback questionnaire, and follow-up questionnaires at 1 month and 3 months postintervention.

Potential participants read the study information in *Writing for Health* and provided informed consent online (by checking a box) to participate. The study was described as investigating whether the writing exercises in the *Writing for Health* program improve the mental and physical well-being of people with diabetes. Both types of writing exercises were described to potential participants. However, the research hypotheses were not revealed.

Potential participants completed automated screening questionnaires, which also provided baseline data. Excluded applicants received an onscreen message informing them that the program is not suitable for them, with links to appropriate resources. All potential participants were provided with online feedback on the severity of their depression and anxiety symptoms.

Participants who met the eligibility criteria proceeded to online registration with the program, completed further online questionnaires (for further baseline data, see below), and were randomized to one of two online writing conditions (BFW or CW). Randomization (1:1) was automatically generated by *Writing for Health* after participants registered with the program. Although the randomization process was concealed from the researchers, they were not blinded to the condition of each participant. At the conclusion of the 3-day writing intervention, participants were invited to complete an online *Feedback Questionnaire*.

Automated reminder emails were sent by *Writing for Health* to participants on each day of their 3-day writing intervention and when it was time to complete their follow-up questionnaires at 1 month and 3 months postintervention. Participants were also provided automated feedback on the severity of their depression and anxiety symptoms at their online follow-up assessments.

### Safety Protocol

Direct contact between participants and researchers did not occur in the standard course of the trial. However, in accordance with the study safety protocol [61], a study psychologist contacted a participant by email or telephone if he/she indicated distress after a writing session or scored in the severe range for depression or anxiety at the 1-month or 3-month follow-up, or indicated possible suicidal thoughts, to assess any need for psychological support and refer the participant to appropriate services, if required.

### Intervention

The online *Writing for Health* website was developed for this study by mental health researchers (including psychologists and a psychiatrist) at St Vincent’s Hospital, Sydney, Australia, and the University of New South Wales, and was hosted on a secure server at the university. Feedback on the usability of the website was previously provided by five adults with T1DM or T2DM during the development process.

Both the BFW and CW conditions involved participants in three 15-minute online writing sessions (once per day for 3 consecutive days), according to the instructions provided. Participants from both conditions continued to receive usual care from their health services.

#### Intervention Condition: Internet-Based Benefit-Finding Writing for Diabetes

Participants in the BFW condition were asked to write about any *positive* thoughts and feelings that they had had about their experiences with diabetes. The instructions (Multimedia Appendix 2) were adapted from those used by Stanton and colleagues (2002) in BFW for women with breast cancer [57]. The same instructions were provided for all three writing sessions, consistent with previous studies of BFW [57,58].

#### Control Condition: Internet-Based Use-of-Time Writing (Control Writing)

Participants in the CW condition were asked to write in detail about how their time was spent that day (first writing session) and the plans for how their time will be spent the following day (second writing session) and week (third writing session). Participants were instructed to be as objective as possible and to focus on the facts and details of how their time was spent (or will be spent), and not to focus on their emotions. These CW instructions were adapted from the control conditions used in previous trials of therapeutic writing [51,56,64,65].

### Measures

#### Primary Outcome Measures

The 17-item Diabetes Distress Scale (DDS17) [61] is a self-report measure of psychosocial stress associated with diabetes, with four reliable subscales: Emotional Burden (feeling overwhelmed by diabetes), Physician-Related Distress (worries about access, trust, and care), Regime-Related Distress (concerns about diet, physical activity, and medications), and Interpersonal Distress (not receiving understanding and appropriate support from others). Cut-off points on the DDS17 have been established for little or no distress, moderate distress, and high distress [65].

The 17-item Benefit Finding Scale [45] was originally developed to investigate benefit finding in women with early stage breast cancer. In the current study, the stem question is modified from, “Having had breast cancer has...” to “Having had diabetes has...” Participants were asked to respond to each of the 17 perceived benefits, such as “has lead me to be more accepting of things” and “has brought my family closer together” on a 5-point scale: 1 - *not at all*, 2 - *a little*, 3 - *moderately*, 4 - *quite a bit*, and 5 - *extremely*. This scale has previously been adapted for use in diabetes (with one item removed) and found to have one large factor and good internal consistency (Cronbach alpha=0.89) in a population of adolescents with T1DM [50].

#### Secondary Outcome Measures

The PHQ-9 [66] was used in the initial online screening to assess current symptoms of depression. It is a brief, widely used, reliable, valid 9-item self-report measure of both severity of depression over the past 2 weeks and is used to make a Diagnostic and Statistical Manual of Mental Disorders-IV criteria–based diagnosis of depression. It has established cut-off scores of 5, 10, 15, and 20, representing mild, moderate, moderately severe, and severe depression, respectively. The total score ranges between 0 and 27, with scores ≥10 having a sensitivity of 88% and a specificity of 88% for major depression [66].

The GAD-7 [67] was used in the initial online screening to assess the current symptoms of anxiety. It is a brief, widely used, reliable, and valid 7-item self-report measure of the severity of anxiety. Scores range from 0 to 21; scores of 5, 10, and 15 represent mild, moderate, and severe anxiety symptoms, respectively. A total score of 8 has been identified as an important threshold for identifying the presence of an anxiety disorder [68].

The Summary of Diabetes Self-Care Activities (SDSCA) measure (revised) is an 11-item self-report measure of self-care of diabetes (including diet, exercise, blood sugar testing, foot care, and smoking) widely used both clinically and in research [69]. Items in the revised version were selected based on their psychometric properties, sensitivity to change, and ease of scoring and interpretation [69]. In a critical appraisal of 26 different measures of diabetes outcomes, the SDSCA measure (revised) was one of only three measures that meets all the criteria of suitability, validity, reliability, and sensitivity to change [70].

Self-rated health was assessed by the question, “In general, how would you rate your health at present?” The five response options were: *very good*, *good*, *fair*, *poor,* and *very poor*. Responses to this question have previously been found to be significantly associated with the blood glucose indicator HbA_1c_ (poorer self-rated health associated with higher HbA_1c_ levels) and number of self-reported diabetes-related symptoms in patients with T2DM [71].

Health care utilization was assessed by the question “In the past month, how many times have you visited a doctor or other health care professional?” at three time-points: baseline, 1-month follow-up, and 3-month follow-up.

#### Additional Measures

The measures below were assessed to conduct manipulation checks.

##### International Positive and Negative Affect Schedule—Short Form

The International Positive and Negative Affect Schedule Short Form (I-PANAS-SF) is a reliable and valid 10-item measure of positive and negative affect, comprised of 10 words that represent positive and negative affect [67]. The values of the correlations between this short-form and the positive and negative affect scales of the full 20-item form of the PANAS are 0.92 and 0.95, respectively [72]. Instructions were modified to assess state rather than trait affect, using the instructions of the 20-item PANAS - Immediate Version [73]. Participants were instructed to indicate the degree of specific affect they feel “right now, at the present moment” on a scale of 1 to 5 (1=very slightly/not at all, 5=extremely).

##### Essay Evaluation Measure

The Essay Evaluation Measure is a 3-item measure adapted from previous studies [57,74], which asks participants to rate, immediately after each writing session, how meaningful, personal, and distressing their writing exercise was, on a 7-point scale (0=not at all, 6=extremely). Similar questions have been used as manipulation checks in previous therapeutic writing studies [57,74]. Immediately after each writing session, participants in the BFW intervention condition were also asked if they were able to identify *any* positive thoughts or feelings about living with diabetes in their writing session.

##### Feedback Questionnaire

The Feedback Questionnaire is a 12-item self-report measure developed to assess participants’ experiences and perceptions of the *Writing for Health* program. Item content was informed by self-report measures from other evaluations of internet-based interventions [75,76] and included its usability, ease of use, credibility, and most and least helpful aspects.

##### Other Measures

Sociodemographic information (age, gender, education, and occupation) and diabetes-related information (type, duration of illness, management, and complications) were also collected.

### Data Analysis

Statistical analyses were conducted using SPSS 22 (IBM Corp, Armonk, NY). Group differences in baseline characteristics were analyzed with two-sample *t* tests and Chi-square tests. The 1-month and 3-month follow-up data were analyzed on an intention-to-treat (ITT) basis using the SPSS mixed procedure. Time was treated as a repeated measures factor (3 levels: T1, T2, T3), with an unstructured residual variance-covariance matrix, and with fixed effects for group, time, and time-by-group interactions. This procedure allowed inclusion of individuals with missing data at follow-up assessments [77].

Planned contrasts were used to examine differences between the groups in changes in outcome variables between baseline and each of the two follow-up occasions and changes over time within groups. All effects were tested at *P*<.05. Within-group and between-group Cohen *d* effect sizes for the primary and secondary outcome measures were also calculated.

Prior to outcome analyses, the distribution of variables was examined for skewness, with variables transformed (log or square root) as needed to reduce skewness to between -1 and 1. All four DDS17 subscale scores and the Blood Sugar Testing subscale of the SDSCA required log transformation; the DDS17, PHQ-9 and GAD-7 total scores required square root transformation. Outliers (scores more than 3 SDs above the mean) were addressed using the winsorizing technique, in which outliers are replaced with the mean value plus 3 SDs [78]. Winsorizing is recommended to reduce the impact of outliers on the Type 1 error rate [78]. Only one outlier value was identified and winsorized.

In addition, manipulation check analyses were conducted to validate the writing intervention instructions in three ways:

- Scores on the Essay Evaluation Measure were compared between the two groups using analysis of variance (ANOVA).
- To examine immediate emotional responses to the writing interventions, scores on the I-PANAS-SF [72] administered immediately before and after each writing session were analyzed using a 2 (group) x 3 (session) x 2 (positive affect and negative affect) repeated measures multivariate ANOVA.
- The content of the written scripts in both groups was assessed using the Linguistic Inquiry Word Count 2015 software program (2015) [79] to examine for differences in positive emotion words and cognitive insight words. This validated method provides a content analysis of the language used in the scripts, quantifying the number of words used from specific categories (eg, emotions and cognitive processes) and has previously been used in numerous studies of therapeutic writing [51,59,80-82]. Linguistic Inquiry Word Count scores for word categories were compared between the two groups using ANOVAs.

### Sample Size and Power Analysis

For this preliminary study, we powered the study to detect a moderately large effect size (Cohen *d*) of 0.7, consistent with previous BFW studies [52,57]. Based on a statistical power of 0.80 and a probability level (Cronbach alpha) of .05, a sample size of 34 per group (ie, 68 for the two groups) was needed for two-tailed tests to detect an effect size (Cohen *d*) of 0.7. Given the expected attrition rate of approximately 34% [41], our target total sample size was 104.

However, the unexpected difficulty encountered in recruiting participants to the trial caused extensive delays in the study timeline. Many different methods were employed to recruit the required sample size, and the recruitment period was extended to assist the effort. Yet, by the end of the extended recruitment period, only 88 of the 104 participants had been recruited (BFW: n=37; CW: n=51). Of these, 72 participants (BFW: n=24; CW: n=48) had completed their baseline measures and were therefore eligible for inclusion in ITT analyses. Revised power analyses were conducted to determine the effect sizes that could be detected with the obtained sample size. Moreover, the unintended unequal allocation of participants eligible for analyses in the two groups (resulting in a 2:1 ratio) would further reduce the statistical power, as a 2:1 ratio requires 12% more participants than a trial using 1:1 to detect the same effect size with equivalent power [83]. Therefore, for ITT analyses in this study (BFW: n=24; CW: n=48), moderately large effect sizes of Cohen *d* ≥0.73 could be detected by two-tailed tests, given a statistical power of 0.80 and a Cronbach alpha of .05.

## Results

### Participant Recruitment and Attrition

**Figure 2:**
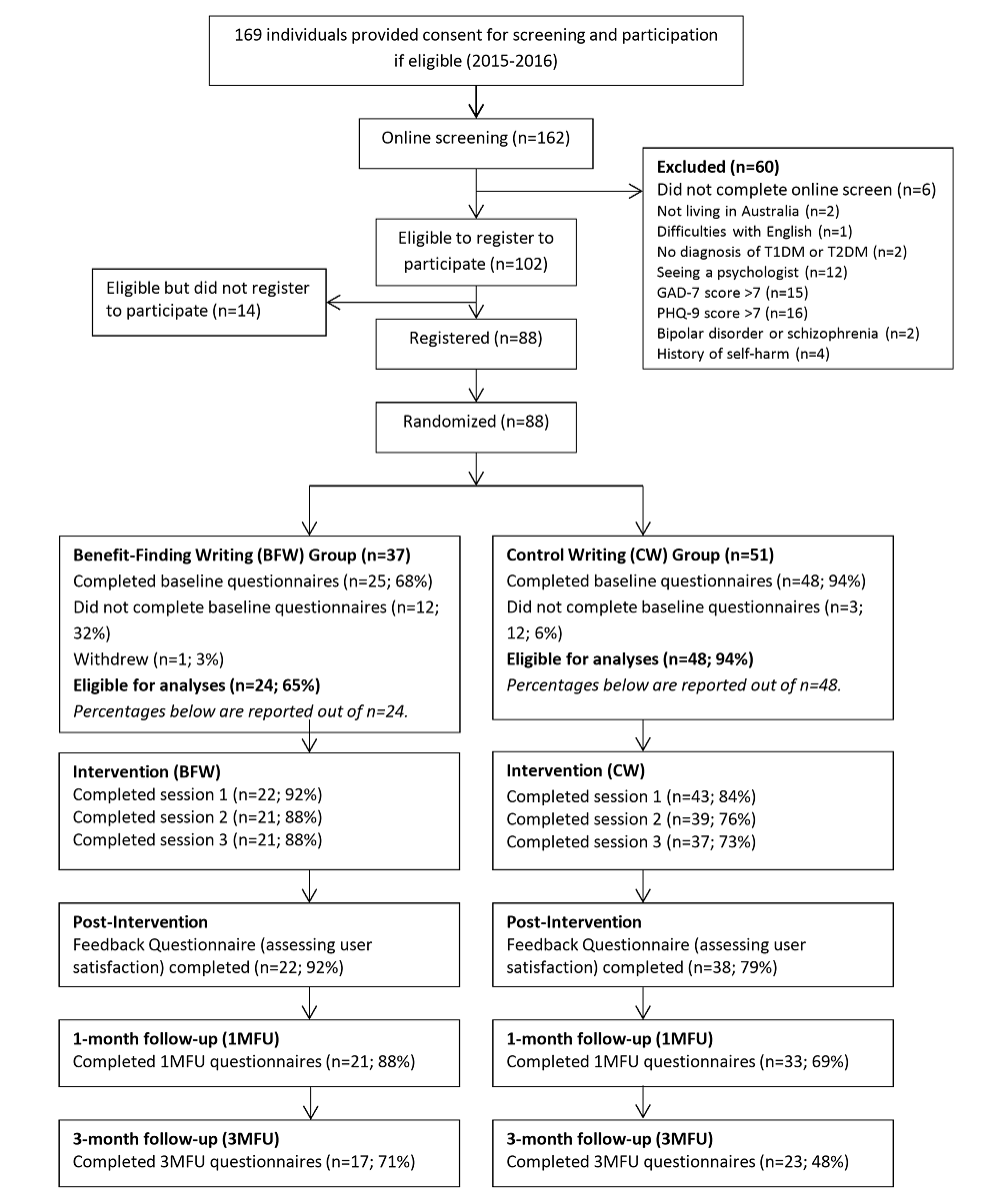
Participant flow diagram. GAD-7: Generalized Anxiety Disorder-7 items; PHQ-9: Patient Health Questionnaire-9 items; T1DM: type 1 diabetes mellitus; T2DM: type 2 diabetes mellitus.

A participant flow diagram is displayed in Figure 2. Over a 21-month period, only 169 individuals provided consent to commence the screening procedure during that period. Of these, 162 commenced the online screening procedure and 102 were eligible to participate, 88 of whom proceeded to online registration and were randomized to either the BFW group (n=37) or the CW group (n=51). Of these participants, 25 allocated to the BFW group and 48 allocated to the CW group completed baseline assessment. One participant in the BFW group formally withdrew from the study after completing the baseline questionnaires but before commencing the intervention. Thus, 24 participants in the BFW group and 48 in the CW group were eligible for inclusion in the ITT analyses (N=72).

At the 1-month follow-up, 21 BFW participants and 33 CW participants completed the questionnaires, while at the 3-month follow-up, 17 BFW participants and 23 CW participants completed the questionnaires. Within the BFW group, 21 participants completed all three writing sessions (88% of those who completed baseline data and 95% those who completed the first writing session). In comparison, 37 of the CW participants completed all three writing sessions (77% of those who completed baseline data and 86% of those who completed the first writing session). There was no significant difference between the BFW group (95%) and the CW group (86%) in the proportion of participants who completed all three writing sessions, of those who commenced the first session (χ^2^_1_=1.341, *P*=.25).

### Baseline Characteristics

Table 1 displays the demographic and clinical baseline characteristics, and Table 2 shows the outcome measures at baseline. A total of 46 participants (64% of the sample) had T1DM and the remaining 26 participants (36%) had T2DM. The majority were women (n=57, 79%) and highly educated, with 63% (n=45) reporting that they had a university degree. Over one-third of the sample was retired (n=27, 38%). The mean response score of 3.96 (SD 0.82) on the current health item (ranging from 1=very poor to 5=very good) corresponded to “good” self-reported current health.

**Table 1:** Participant demographic and baseline characteristics in the benefit-finding writing and control writing groups and the total sample.

Characteristic			Total sample (N=72)	BFW^a^ (n=24)	CW^b^ (n=48)	*P* value^c^
**Demographics**						
	Age (years), mean (SD)		53.79 (15.93)	53.54 (17.26)	53.92 (15.41)	.80
	Female, n (%)		57 (79)	20 (83)	37 (77)	.64
	Married/de facto, n (%)		44 (62)	15 (65)	29 (60)	.51
	Employed (full-time or part-time), n (%)		30 (42)	9 (39)	21 (44)	.40
	Retired, n (%)		27 (38)	10 (44)	17 (35)	.29
	University educated, n (%)		45 (63)	14 (61)	31 (65)	.72
**Clinical characteristics**						
	Type 1 diabetes, n (%)		46 (64)	15 (62)	31 (65)	.87
	Years since diabetes diagnosis, mean (SD)		17.69 (14.26)	16.86 (13.68)	18.08 (14.65)	.78
	Diabetes complications, n (%)		16 (22)	4 (17)	12 (22)	.69
	**Diabetes management^d^, n (%)**					
		Insulin	51 (71)	17 (71)	34 (71)	.98
		Medication	24 (33)	6 (25)	18 (38)	.60
		Blood sugar testing	67 (93)	24 (100)	43 (90)	.68
		Healthy eating plan	59 (82)	18 (75)	41 (85)	.73
		Exercise	54 (75)	16 (67)	38 (79)	.61
	Current antidepressant medication, n (%)		13 (18)	6 (25)	7 (15)	.24
	Ever been depressed, n (%)		33 (46)	12 (50)	21 (44)	.50
	Ever had a mental illness diagnosed, n (%)		22 (31)	7 (29)	15 (31)	.88

^a^BFW: benefit-finding writing.

^b^CW: control writing.

^c^*P* values for variables based on means are from a two-sample *t* test. *P* values for variables based on percentages are from a Chi-square test.

^d^Categories were not mutually exclusive and participants could indicate more than one management strategy to best describe their situation.

As reported in Table 2, participants’ baseline levels of depression, anxiety, and diabetes distress were very low. Baseline scores on the PHQ-9 (mean 2.25, SD 1.62) indicated that most participants were in the “minimal to none” range (0 to 4) for depression symptoms [62]; similarly, baseline scores on the GAD-7 (mean 1.71, SD 1.73) indicated that most participants were in the “minimal to none” range (0 to 4) for anxiety symptoms [63]. Further, baseline scores on the DDS (mean 1.74, SD 0.66) and all four subscales of the DDS17 (means ranging from 1.65 to 1.95) were in the range of “DDS < 2.0: little or no diabetes distress” [65]. However, almost one half of the sample (n=33, 46%) reported ever experiencing depression for 2 weeks or more in the past (Table 1).

There were no significant differences between the intervention (BFW) and control (CW) groups in any baseline characteristics (demographics, clinical characteristics, or outcome measures), with the exception of a single item of the SDCSA measure (revised) assessing fruit and vegetable consumption (*t*_70_=2.484, *P*=.02). The CW group had a significantly higher mean number of days (5.58 days, SD 1.41 days) than the BFW group (4.30 days, SD 2.34 days) in which they ate five or more serves of fruits and vegetables over the past 7 days.

**Table 2:** Baseline scores on primary and secondary outcome measures in the benefit-finding writing and control writing groups and the total sample.

Measure			Total sample (N=72)	BFW^a^ (n=24)	CW^b^ (n=48)	*P* value^c^
**Primary outcome measures**						
	Benefit Finding Scale, mean (SD)		43.81 (16.67)	39.83 (17.16)	45.79 (16.23)	.45
	**Diabetes Distress Scale, mean (SD)**					
		Total score	1.73 (0.64)	1.74 (0.66)	1.73 (0.63)	.60
		Emotional Burden	1.65 (0.68)	1.66 (0.66)	1.65 (0.70)	.49
		Physician-Related Distress	1.68 (0.71)	1.70 (0.70)	1.67 (0.71)	.39
		Regimen-Related Distress	1.73 (0.66)	1.80 (0.71)	1.69 (0.64)	.67
		Interpersonal Distress	1.95 (0.87)	1.83 (0.77)	2.01 (0.92)	.56
**Secondary outcome measures**						
	PHQ-9^d^, mean (SD)		2.11 (1.59)	2.25 (1.62)	2.04 (1.58)	.31
	GAD-7^e^, mean (SD)		1.60 (1.71)	1.71 (1.73)	1.54 (1.71)	.24
	**Revised Summary of Diabetes Self-Care Activities**					
		General Diet, mean (SD)	5.29 (1.68)	4.93 (2.14)	5.46 (1.41)	.86
		Specific Diet—Fruit and Veg, mean (SD)	5.17 (2.10)	4.30 (2.34)	5.58 (1.87)	.02^f^
		Specific Diet—High-Fat Foods, mean (SD)	3.39 (2.16)	3.61 (2.45)	3.29 (2.03)	.56
		Exercise, mean (SD)	3.79 (2.26)	3.39 (2.28)	3.98 (2.25)	.45
		Blood Glucose Testing, mean (SD)	5.54 (2.28)	5.50 (2.27)	5.56 (2.31)	.76
		Foot Care, mean (SD)	2.53 (2.52)	2.17 (2.67)	2.70 (2.45)	.19
		Smoking Status, n (%)	7 (10)	1 (4)	6 (13)	.23
	Self-reported health, mean (SD)		3.96 (0.82)	4.00 (0.80)	3.94 (0.84)	.78
	Number of visits to a health professional in the past 30 days, mean (SD)		1.82 (2.17)	2.04 (1.97)	1.71 (2.28)	.56

^a^BFW: benefit-finding writing.

^b^CW: control writing.

^c^*P* values for variables based on means are from a two-sample *t* test. *P* values for variables based on percentages are from a Chi-square test.

^d^PHQ-9: Patient Health Questionnaire-9 items.

^e^GAD-7: Generalized Anxiety Disorder-7 items.

^f^Statistically significant at *P*<.05.

### Manipulation Checks

#### Essay Evaluation Measure

Consistent with predictions, participants in the BFW group rated their writing exercises as significantly more personal (*F*_1,56_=6.00, *P*=.02, *Ƞ*_p_^2^=0.09) and significantly more meaningful (*F*_1,56_=6.87, *P*=.01, *Ƞ*_p_^2^=0.11) than those in the CW group. The two groups did not differ significantly in terms of the ratings of how distressing the writing exercises were (*F*_1,56_=2.76, *P*=.10, *Ƞ*_p_^2^=0.05).

The majority of participants in the BFW group reported being able to identify positive thoughts or feelings about diabetes in their writing sessions: 20 (91%) in Writing Session 1, 21 (100%) in Writing Session 2, and 20 (95%) in Writing Session 3.

#### Linguistic Content Analyses

Consistent with predictions, participants in the BFW group used a significantly greater proportion of positive emotion words (*F*_1,56_=128.37, *P*<.001, *Ƞ*_p_^2^=0.70), negative emotion words (*F*_1,56_=55.23, *P*<.001, *Ƞ*_p_^2^=0.50), causal words (*F*_1,56_=49.71, *P*<.001, *Ƞ*_p_^2^=0.47), and cognitive insight words (*F*_1,56_=180.32, *P*<.001, *Ƞ*_p_^2^=0.76) than those in the CW group. The two groups did not differ in the total number of words used per session (*F*_1,56_=0.13, *P*=.72, *Ƞ*_p_^2^=0.00).

**Figure 3:**
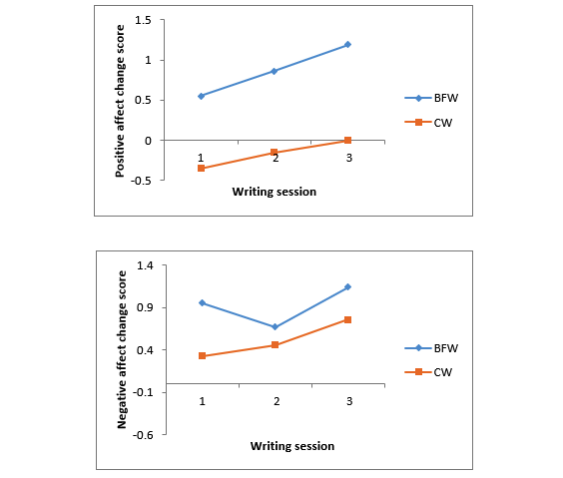
Mean positive and negative affect change scores on the International Positive and Negative Affect Schedule - Short Form in the benefit-finding writing and control writing groups. BFW: benefit-finding writing; CW: control writing.

#### Positive and Negative Affect

Change scores (post-pre) for the Positive Affect and Negative Affect subscales of I-PANAS-SF were computed for each of the three writing sessions (Figure 3). As predicted, the BFW group had significantly greater increases in positive affect than the CW group (*F*_1,56_=7.76, *P*=.01 *, Ƞ*_p_^2^=0.12). The increases in negative affect following writing did not differ significantly between the BFW and CW groups (*F*_1,56_=2.37, *P*=.13 *, Ƞ*_p_^2^=.04).

### Effect of Writing Interventions on Primary Outcome Measures

#### Between-Group Differences in Primary Outcome Measures at 1-Month and 3-Month Follow-Ups

**Figure 4:**
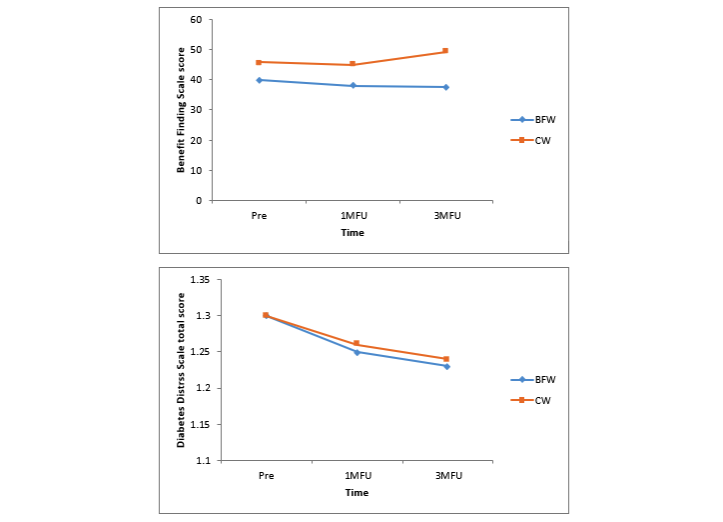
Estimated means of the primary outcome measures in the benefit-finding writing and control writing groups at baseline (pre), 1-month follow-up, and 3-month follow-up using the Benefit Finding Scale and Diabetes Distress Scale. 1MFU: 1-month follow-up; 3MFU: 3-month follow-up; BFW: benefit-finding writing; CW: control writing.

Table 3 presents the observed means and estimated marginal means for the primary outcome measures at baseline and 1-month and 3-month follow-ups. Table 4 reports the between-group results of the SPSS mixed procedure analyses and the Cohen *d* effect sizes. Figure 4 displays the estimated means of the primary outcome measures in the two groups at baseline, 1-month follow-up, and 3-month follow-up.

There were no significant group-by-time interactions in the mixed model analyses for the BFS or DDS17 scores (*F*_2,45.75_=2.18, *P*=.12 and *F*_2,45.75_=0.34, *P*=.97, respectively). As displayed in Table 4, there were no significant group-by-time interactions for any of the planned contrasts examining differences between the BFW and CW groups in changes from baseline to 1-month follow-up or 3-month follow-up for the BFS, total DDS17 score, or any of the four subscales of the DDS17 (all *P*>0.05). In addition, as displayed in Table 4, all between-group Cohen *d* effect sizes for the primary outcomes were minimal to small.

**Table 3:** Results of primary outcome measures: observed and estimated means with SDs at baseline and 1-month and 3-month follow-ups.

Primary outcome for each group			Observed means (SD)			Estimated means^a^ (SD)		
			Baseline^b^	1-month follow-up^c^	3-month follow-up^d^	Baseline	1-month follow-up	3-month follow-up
**Benefit Finding Scale**								
	BFW^e^		39.83 (17.16)	39.91 (18.68)	40.06 (18.22)	39.83 (16.52)	38.10 (18.78)	37.61 (19.07)
	CW^f^		45.79 (16.23)	46.97 (19.12)	49.09 (19.77)	45.79 (16.56)	45.04 (19.61)	49.29 (20.58)
**Diabetes Distress Scale Total**								
	BFW		1.74 (0.66)	1.62 (0.56)	1.54 (0.65)	1.30 (0.24)	1.25 (0.20)	1.23 (0.24)
	CW		1.73 (0.63)	1.67 (0.57)	1.57 (0.48)	1.30 (0.21)	1.26 (0.21)	1.24 (0.28)
**Diabetes Distress Scale Subscales**								
	**Emotional Burden**							
		BFW	1.66 (0.66)	1.75 (0.81)	1.58 (0.62)	0.44 (0.39)	0.48 (0.44)	0.42 (0.39)
		CW	1.65 (0.70)	1.83 (0.77)	1.79 (0.61)	0.43 (0.34)	0.51 (0.48)	0.52 (0.48)
	**Physician-Related Distress**							
		BFW	1.70 (0.70)	1.42 (0.91)	1.44 (0.81)	0.27 (0.20)	0.12 (0.20)	0.15 (0.20)
		CW	1.67 (0.71)	1.34 (0.49)	1.26 (0.41)	0.27 (0.21)	0.14 (0.21)	0.11 (0.28)
	**Regimen-Related Distress**							
		BFW	1.80 (0.71)	1.74 (0.69)	1.58 (0.57)	0.52 (0.34)	0.48 (0.34)	0.42 (0.39)
		CW	1.69 (0.64)	1.67 (0.66)	1.57 (0.63)	0.47 (0.28)	0.43 (0.35)	0.40 (0.35)
	**Interpersonal Distress**							
		BFW	1.83 (0.77)	1.46 (0.59)	1.55 (1.02)	0.39 (0.29)	0.24 (0.29)	0.21 (0.34)
		CW	2.01 (0.92)	1.87 (1.07)	1.59 (0.89)	0.43 (0.28)	0.32 (0.35)	0.27 (0.35)

^a^Estimated means based on intention-to-treat sample of benefit-finding writing (n=24) and control writing (n=48), with variables transformed (log or square root) to address skewness, SDs, and CIs shown in parentheses.

^b^Benefit-finding writing: n=24, Control writing: n=48.

^c^Benefit-finding writing: n=21, Control writing: n=33.

^d^Benefit-finding writing: n=17, Control writing: n=23.

^e^BFW: benefit-finding writing.

^f^CW: control writing.

**Table 4:** Estimated differences in mean change of primary outcomes between baseline and 1-month or 3-month follow-up for the benefit-finding writing and control writing groups.

Outcome		Baseline to 1-month follow-up^a^			Baseline to 3-month follow-up^b^		
		Estimated mean difference (95% CI)	*P* value	Cohen *d* (95% CI)	Estimated mean difference (95% CI)	*P* value	Cohen *d* (95% CI)
Benefit Finding Scale		0.98 (–5.19 to 7.15)	.32	–0.06 (–0.63 to 0.50)	5.72 (–0.52 to 11.97)	.07	–0.34 (–0.80 to 0.19)
Diabetes Distress Scale, total score		0.01 (–0.07 to 0.09)	.82	–0.05 (–0.54 to 0.44)	0.01 (–0.06 to 0.09)	.83	–0.05 (–0.53 to 0.45)
**Diabetes Distress Scale subscales**							
	Emotional Burden	0.05 (–0.16 to 0.25)	.65	–0.11 (–0.59 to 0.39)	0.11 (–0.07 to 0.29)	.23	–0.31 (–0.79 to 0.20)
	Physician-Related Distress	0.02 (–0.10 to 0.13)	.74	–0.10 (–0.59 to 0.38)	–0.05 (–0.15 to 0.06)	.38	0.19 (–0.31 to 0.67)
	Regimen-Related Distress	–0.00 (–0.12 to –0.11)	.95	–0.02 (–0.49 to 0.47)	0.02 (–0.11 to 0.16)	.74	–0.10 (–0.58 to –0.40)
	Interpersonal Distress	0.04 (–0.11 to 0.19)	.61	–0.14 (–0.63 to 0.36)	0.01 (–0.14 to 0.16)	.88	–0.07 (–0.56 to 0.42)

^a^Difference = (baseline – 1-month follow-up [for benefit-finding writing]) – (baseline – 1-month follow-up [for control writing]).

^b^Difference = (baseline – 3-month follow-up [for benefit-finding writing]) – (baseline – 3-month follow-up [for control writing]).

#### Within-Group Differences in Primary Outcome Measures at 1-Month and 3-Month Follow-Ups

Table 5 displays the results of mixed procedure analyses for within-group changes on the primary outcomes from baseline to 1-month and 3-month follow-ups. There were no significant within-group changes from baseline in terms of benefit finding in relation to diabetes (BFS scores). However, significantly lower diabetes distress (DDS17 total score) was observed in both groups at the 3-month follow-up, but not the 1-month follow-up, compared to the baseline. The within-group changes in the four subscales of DDS17 were also examined. Both the BFW and CW groups displayed significant within-group reductions in two subscales of the DDS17 (Physician-Related Distress and Interpersonal Distress) at both 1-month and 3-month follow-ups. As shown in Table 5, medium effect sizes were observed for these within-group DDS17 subscale changes. However, neither group displayed any significant within-group changes in the other two subscales of the DDS17 (Emotional Burden and Regimen-Related Distress).

**Table 5:** Within-group estimated changes in primary outcomes between baseline and 1-month and 3 month follow-ups.

Outcome and group			Baseline to 1-month follow-up^a^			Baseline to 3-month follow-up^b^		
			Estimated mean difference (95% CI)	*P* value	Cohen *d* effect size*d* (95% CI)	Estimated mean difference (95% CI)	*P* value	Cohen *d* effect size *d* (95% CI)
**Benefit Finding scale**								
	BFW^c^		1.74 (–3.13 to 6.60)	.48	0.10 (–0.47 to 0.66)	2.22 (–2.59 to 7.03)	.48	0.12 (–0.44 to 0.69)
	CW^d^		0.76 (–3.04 to 4.55)	.69	0.04 (–0.36 to 0.44)	–3.50 (–7.48 to 0.48)	.08	–0.19 (–0.59 to 0.21)
**Diabetes Distress Scale total**								
	BFW		0.05 (–0.20 to 0.11)	.14	0.23 (–0.58 to 1.03)	0.07 (0.01 to 0.13)	.03	0.29 (–0.51 to 1.10)
	CW		0.04 (–0.01 to 0.08)	.13	0.19 (–0.38 to 0.76)	0.06 (0.01 to 0.11)	.02	0.24 (–0.33 to 0.81)
**Diabetes Distress Scale subscales**								
	**Emotional Burden**							
		BFW	–0.04 (–0.20 to 0.13)	.65	–0.10 (–0.90 to 0.70)	0.02 (–0.11 to 0.16)	.75	0.05 (–0.75 to 0.85)
		CW	–0.08 (–0.21 to 0.04)	.18	–0.19 (–0.76 to 0.38)	–0.09 (–0.20 to 0.03)	.13	–0.22 (–0.78 to 0.35)
	**Physician-Related Distress**							
		BFW	0.15 (0.06 to 0.24)	<.001	0.75 (–0.08 to 1.58)	0.11 (0.03 to 0.20)	.007	0.60 (–0.22 to 1.42)
		CW	0.13 (0.06 to 0.20)	<.001	0.62 (–0.04 to 1.20)	0.16 (0.09 to 0.22)	<.001	0.65 (–0.53 to 1.23)
	**Regimen-Related Distress**							
		BFW	0.03 (–0.06 to 0.13)	.49	0.12 (–0.68 to 0.92)	0.09 (–001 to 0.20)	.08	–0.27 (–1.08 to 0.53)
		CW	0.03 (–0.04 to 0.11)	.36	0.13 (–0.44 to 0.69)	0.07 (–0.02 to 0.16)	.11	–0.22 (–0.79 to 0.35)
	**Interpersonal Distress**							
		BFW	0.15 (0.03 to 0.27)	.02	0.52 (–0.30 to 1.33)	0.17 (0.06 to 0.29)	.004	0.57 (–0.25 to 1.39)
		CW	0.11 (0.02 to 0.22)	.02	0.35 (0.22 to 0.95)	0.16 (0.07 to 0.26)	<.001	0.51 (0.07 to 1.08)

^a^Change = baseline – 1-month follow-up.

^b^Change = baseline – 3-month follow-up.

^c^BFW: benefit-finding writing.

^d^CW: control writing.

### Effect of Writing Interventions on Secondary Outcome Measures

#### Between-Group Differences in Secondary Outcome Measures at 1-Month and 3-Month Follow-Ups

Multimedia Appendix 3 includes the observed means and estimated marginal means for the secondary outcome measures at baseline and follow-ups. The results for the between-group differences in secondary outcomes are displayed in Multimedia Appendix 4. There was only one significant group-by-time interaction for any of the secondary outcome measures examined. For self-reported health, there was a significant difference between the BFW and CW groups in changes from baseline to 1-month follow-up (*P*=.04), reflecting an increase in self-reported health in the CW group and a decrease in the BFW group (neither of these within-group changes were significant). However, there was no significant difference between the two groups in changes in self-reported health from baseline to the 3-month follow-up (*P*=.81). There were no significant group-by-time interactions for scores of the PHQ-9, GAD-7, revised SDSCA, or health care utilization.

#### Within-Group Differences in Secondary Outcome Measures at 1-Month and 3-Month Follow-Ups

The results for the within-group differences in secondary outcomes are displayed in Multimedia Appendix 5. Both the BFW and CW groups displayed significant increases in depression (PHQ-9 scores) and anxiety (GAD-7) scores over time. In both the BFW and CW groups, there were significant within-group increases in both the PHQ-9 and GAD-7 scores from baseline to 1-month follow-up and a significant increase in GAD-7 scores from baseline to the 3-month follow-up. The CW group also displayed a significant increase in PHQ-9 scores from baseline to the 3-month follow-up. The effect sizes for these within-group changes ranged from small to moderately large. The observed mean PHQ-9 and GAD-7 scores in both groups at both follow-ups remained in the nonclinical range (2.79 to 4.47), which is in the “none-minimal” range (<5) for depression and anxiety severity.

There were few other significant within-group changes in the secondary outcomes. In the BFW group, there was a significant decrease in Exercise scores on the revised SDSCA at the 1-month follow-up, but not at the 3-month follow-up. In the CW group, there was a significant increase in the Foot Care subscale of the revised SDSCA at the 1-month follow-up, but not at the 3-month follow-up. There were no significant within-group changes over time in either group for the General Diet, Specific Diet, or Blood Glucose Testing subscales of the revised SDSCA or self-reported health or health care utilization.

### Safety

Mean distress ratings reported after each session were low for both groups in all three writing sessions, ranging from 0.14 to 1.05. Two participants in the BFW group (8%) and two in the CW group (4%) were contacted by email following a high distress rating (≥5/6); all four participants reported that their distress was short-lived. Four participants in the BFW group (17%) and two in the CW group (4%) were contacted by telephone due to elevated scores on the PHQ-9 or GAD-7 at follow-up(s), in line with the study safety protocol [61]. One participant (in the BFW group) was referred to a mental health service after reporting a recurrence of previous depression at the 3-month follow-up. All six participants contacted by telephone had reported a history of depression or a diagnosis of a mental illness at baseline. There were no privacy breaches or technical difficulties during the trial.

### Acceptability

The BFW group (n=22) and CW group (n=38) did not differ in their mean total score for the Feedback Questionnaire (BFW: mean 16.18, SD 4.89; CW: mean 15.36, SD 5.34; *t*_58_=0.59, *P*=.56). In the BFW group, 71% (n=15) of the participants reported that they were “mostly” to “very” satisfied with the *Writing for Health* program compared to 55% (n=21) in the CW group (χ^2^_1_=1.47, *P*=.23). Approximately half of the participants in both the BFW group (n=11, 51%) and CW group (n=18, 47%) reported that they would be “mostly” to “very” confident in recommending *Writing for Health* to a friend with diabetes (χ^2^_1_=0.14, *P*=.71). Further, approximately one-third of both groups (BFW: n=7, 33%; CW: n=13, 34%) reported that the writing exercises were “mostly” to “very” helpful in reducing stress (χ^2^_1_=0.01, *P*=.95).

## Discussion

### Principal Findings

This RCT examined the efficacy of BFW, compared to a CW condition, among adults with T1DM or T2DM who were not currently experiencing MDD or an anxiety disorder. It was hypothesized that participants randomized to the BFW group, compared to those in the CW group, would have significant increases in benefit finding, significant reductions in diabetes distress (primary outcomes), and significant improvements in the secondary outcomes at both the 1-month and 3-month follow-ups. However, these hypotheses were not supported by our results. In addition, there were no significant intervention effects on the primary outcomes.

All the hypotheses for the validation analyses were supported by our results. This suggests that the results for the outcome measures cannot be explained by participants not following the writing instructions or failing to engage with the writing tasks as expected. Specifically, consistent with other BFW studies [52,56,57], participants in the BFW group rated their writing sessions as significantly more personal and meaningful than those in the CW group. Second, as in previous findings [43,84,85], the BFW group had a significantly greater increase in positive affect following writing, relative to the CW group. Third, consistent with previous findings, linguistic analyses revealed that BFW participants had a greater use of positive emotion words [51,85-87], negative emotion words [51,85], and cognitive processing words [51,85,88,89] than CW participants. Further, the majority of BFW participants reported that they were able to identify at least one positive aspect of living with diabetes in their writing session that day. Overall, these findings suggest that the BFW participants followed their writing instructions and, as expected, engaged in both emotional expression and cognitive processing to a greater degree than control group participants. However, there were no significant between-group differences in the primary and secondary outcome variables. A second review of the literature produced several potential explanations.

To our knowledge, this was the first study to examine benefit finding as an outcome of therapeutic writing, so it is not possible to directly compare this finding with previous trials. However, posttraumatic growth, a concept closely related to benefit finding [90], has been found to increase following BFW to a greater degree than following EW [43]. Further, more intensive interventions have previously been found to increase benefit finding in medical populations [91,92]. For example, in adults with T2DM, benefit finding was found to increase following 14 sessions of telephone health coaching [92]. Our BFW intervention (of three 15-minute sessions) was substantially briefer, with less clinician contact. Although the intervention brevity and content were chosen to avoid potential resistance to psychological care by people with diabetes, it is conceivable that a more intensive, or simply more efficacious, intervention is required to increase benefit finding in people with diabetes.

Contrary to our hypotheses, the BFW group did not show significant reductions in diabetes distress, compared to the control group, at either the 1-month or 3-month follow-up. Rather, both groups had significant within-group reductions in diabetes distress, specifically in Physician-Related Distress and Interpersonal Distress, which are related to diabetes-related support from health professionals and family and friends, respectively. Thus, one possibility is that the process of participating in a research study about experiences of living with diabetes, albeit with limited clinician support, was sufficient to increase the level of perceived social support in relation to diabetes, regardless of the writing intervention instructions. This explanation is consistent with the findings of the REDEEM trial, in which three brief online interventions, all accompanied by support telephone calls, resulted in significantly reduced diabetes distress in nondepressed adults with T2DM [30]. As suggested by the authors [30], it appeared that nondepressed adults with diabetes distress were highly responsive to professional attention and normalization of diabetes distress. The finding of a small reduction in diabetes distress in both groups in this study is consistent with the findings of a meta-analysis of interventions for reducing diabetes distress [93], which found that even generalist and psychoeducation interventions resulted in small reductions in diabetes distress in adults with T1DM or T2DM.

Further, the lack of a significant reduction in diabetes distress, compared to a control group, is consistent with the findings of an RCT of standard EW for adults with T2DM [40] and a pilot RCT of EW about stressors occurring in the past month in nondepressed adults with T2DM [41]. It is also in line with the meta-analysis showing that only interventions of six or more sessions resulted in significant reductions in diabetes distress compared to a control group [93]. Thus, overall, it appears that while attention to diabetes distress and a generalist intervention may be sufficient to result in small reductions in diabetes distress in nondepressed adults, a longer or more efficacious intervention is required to provide significant reductions in diabetes distress compared to an active control condition.

This study did not find the predicted improvements in the secondary outcomes of depression, anxiety, diabetes self-care, self-reported health, and health care utilization. Of note, participants in both groups demonstrated significant increases in mean depression and anxiety scores over a period of 3 months, albeit with mean scores remaining in the “none-minimal” range for symptoms. There were no significant between-group differences in these changes. This finding was inconsistent with previous findings of a reduction in symptoms of depression or anxiety following diabetes-specific EW [39], writing about use of time [64], or online positive affect journaling in medical patients with elevated anxiety [44]. Given that the baseline depression and anxiety scores in our study were so low (2.11 and 1.60, respectively), the increase in mean scores may reflect a regression to the mean [94]. The baseline means were unexpectedly substantially below the inclusion criteria cut-off scores of 8 and lower than the normative means of adults with T1DM or T2DM in Australia (5.6 to 7.7 on the PHQ-9 and 4.0 to 5.3 on the GAD-7) [95]. Further, half of the sample reported a history of depression, and it is known that the adults with diabetes and past depression are at an increased risk of a recurrence of depression [96]. A minority of participants with a history of depression experienced worsening of depression or anxiety symptoms over the 3-month study period, which may have accounted for the increase in mean scores in both groups. Regardless of the reason for these changes, this finding highlights the need for monitoring of depression and anxiety in trial participants with diabetes and past depression.

### Safety

Given that one of the key potential benefits of BFW, relative to EW, is the absence of short-term distress that typically accompanies EW [33], it was important for this study to assess immediate emotional responses to BFW in people with diabetes. This study found that participants in the BFW group reported no more distress than those writing about use of time and had greater increases in positive affect immediately after writing. This supports the hypothesis that BFW may be more suitable for online dissemination than forms of therapeutic writing that typically lead to short-term distress [36]. However, participants in this study had few symptoms of depression or anxiety, and it is not known how depressed adults with diabetes would respond to BFW.

### Feasibility of the Intervention

Feasibility of Web-based BFW for adults with T1DM or T2DM was assessed by adherence to and acceptability of the intervention. Adherence to BFW was high, with 95% of those who commenced the first writing session also completing all three writing sessions. Further, manipulation checks indicated that those in the BFW group were able to write about the positive aspects of diabetes and engaged with the intervention as intended. However, acceptability of the intervention was only moderate. Although over two-thirds of the participants in the BFW group reported satisfaction with the *Writing for Health* program, only half of both groups would recommend it to a friend.

Further, given the small sample recruited over a 21-month period, there was no high level of demand for Web-based BFW in adults with diabetes. Other studies of therapeutic writing in participants with diabetes have also reported difficulty in recruiting their target sample sizes [39,41]. This is in line with the observation that participants’ reasons for undertaking unfacilitated therapeutic writing are often unclear, particularly among those with a chronic illness [33].

Thus, this study identified some limitations to the feasibility of Web-based BFW for adults with diabetes, with apparent low interest in the intervention and only moderate acceptability, despite high adherence among those who commenced the writing sessions. Recent qualitative findings (published subsequent to this study) regarding the perceptions of written reflection about T2DM [97] have shed some light on the appropriateness of therapeutic writing for adults with diabetes. Specifically, it was found that while some adults with T2DM reported that writing about diabetes increased their commitment to diabetes self-management, others perceived that a written reflection was inapplicable to their diabetes. Some found that writing about diabetes was difficult, the timing in terms of their disease trajectory or life priorities was inappropriate, and they required a meeting with a diabetes nurse in order to benefit from the task. The authors concluded that written reflection about diabetes may only be suitable for some adults with T2DM and that it may be more appropriate as part of a “blended” approach rather than a standalone intervention [97]. The findings from this study regarding the feasibility of BFW for diabetes support this recommendation.

### Limitations and Future Directions

There were several limitations to this study. A clear limitation was the small sample size, which reduced the statistical power such that only moderately large effect sizes for differences between the two groups, if present, could have been detected. Second, given the preliminary nature of this trial, multiple comparisons of several independent variables were conducted without control of the alpha levels to reduce the risk of Type 1 errors (ie, “false positive”). However, given that there were few significant results, this is unlikely to be a significant issue.

Perhaps, most importantly, the generalizability of the results of this study was limited by the profile of the sample who registered to participate. Although the conservative eligibility criteria were set in line with the exploratory nature of the study, baseline levels of diabetes distress, depression, and anxiety were low, leaving very little room for improvement. It has recently been suggested that therapeutic writing may offer the most benefit for those with moderate levels of symptoms, as those with very few symptoms cannot improve their symptoms with this treatment and those with severe symptoms may require stronger treatment [44]. Therefore, future trials of therapeutic writing in adults with diabetes should apply a minimum threshold for diabetes distress, to examine its efficacy among those with elevated symptoms, as well as the maximum cut-off scores for depression and anxiety to exclude those with a likely mental illness.

Physical symptoms and biological indicators such as HbA_1c_ were not assessed. However, given that physical symptoms tend to be more responsive to therapeutic writing interventions than psychological symptoms [33,35], future studies of therapeutic writing in people with diabetes should include a self-report measure of physical symptoms.

Finally, the current study found that BFW was only moderately acceptable to adults with T1DM or T2DM. Given the reluctance of many people with diabetes to engage in mental health care, future research should examine predictors of engagement with low-intensity interventions, especially those such as BFW, given that it appears to appeal to only some adults with diabetes. Further, since a participatory design is known to increase the acceptability and use of interventions [98], the development of future therapeutic writing interventions should include the participation of adults with diabetes at every design stage. Although this study set out to use BFW because previous research suggested that it may be suitable for online dissemination due to a lack of distress involved [36,51,59], one future direction could be the co-design of a “combined” therapeutic writing, which would allow users to express negative thoughts and feelings about diabetes as well as perceived benefits, with strategies to encourage cognitive change and coping [99,100].

### Conclusions

The results of this preliminary RCT found that Web-based BFW for nondepressed adults with T1DM or T2DM was no more efficacious than a CW condition in improving diabetes distress or benefit finding over a period of 3 months. Possibly due to very low baseline levels of depression and anxiety in the sample, BFW in this study was not efficacious in improving the symptoms of depression or anxiety, diabetes self-care, self-reported health, or health care utilization compared to CW.

BFW was adhered to and was associated with increases in positive affect and no more distress than the control condition. This suggests that BFW for diabetes may be more suitable for online dissemination than traditional EW, which typically results in short-term distress. Hence, future research should continue to investigate the efficacy of BFW for adults with T1DM or T2DM, using a larger sample of participants with elevated diabetes distress. Further, engaging a co-design process may improve the perceived helpfulness of therapeutic writing in adults with diabetes. Despite these issues, further research in this population is warranted, as therapeutic writing offers potential as a simple and cost-effective low-intensity intervention, especially for people with diabetes who may not wish to consider mental health interventions.

## Appendix

Multimedia Appendix 1 CONSORT-EHEALTH checklist (V 1.6.1).

Multimedia Appendix 2 Instructions for Web-based benefit-finding writing for diabetes.

Multimedia Appendix 3 Results of secondary outcome measures: observed and estimated means, with SDs at baseline and 1-month and 3-month follow-ups.

Multimedia Appendix 4 Estimated differences in mean change of secondary outcomes between baseline and 1-month and 3-month follow-ups for the benefit-finding writing group and the control writing group.

Multimedia Appendix 5 Within-group estimated changes in secondary outcomes between baseline and 1-month and 3-month follow-ups.

## Abbreviations

BFW: benefit-finding writing
CW: control condition of online writing
EW: expressive writing
GAD-7: Generalized Anxiety Disorder-7
HbA_1c_: hemoglobin A_1c_
I-PANAS-SF: International Positive and Negative Affect Schedule - Short Form
ITT: intention to treat
MDD: major depressive disorder
PHQ-9: Patient Health Questionnaire-9 items
RCT: randomized controlled trial
SDSCA: Summary of Diabetes Self-Care Activities
T1DM: type 1 diabetes mellitus
T2DM: type 2 diabetes mellitus
