# Supplementary material for: Web-Based Benefit-Finding Writing for Adults with Type 1 or Type 2 Diabetes: Preliminary Randomized Controlled Trial
Source: JMIR Diabetes. 2019 Jun 27;4(2):e13857. doi: 10.2196/13857 (PMC6620889; doi:10.2196/13857)
Supplement: Multimedia Appendix 2 [file diabetes_v4i2e13857_app2.pdf]

Multimedia Appendix 2: Instructions for Internet-based benefit-finding writing for diabetes (BFW)

What we would like you to write about for these three sessions are any **POSITIVE thoughts and feelings about your experience with diabetes.** We realise that people with diabetes experience a full range of emotions that often includes some positive emotions, thoughts, and changes. In this writing exercise we want you to focus only on the positive thoughts and feelings that you have experienced over the course of your diabetes.

We realise that this may be difficult at first, as living with diabetes can be challenging at times. Don't worry if you find it difficult at first to find some positive thoughts and feelings related to diabetes – but please take the time to think about it and try and write about some today.

You might think about some positive feelings and changes that you experienced before being diagnosed with diabetes, after diagnosis, during management, and now. For example, some people feel that they have gained important lessons out of their experiences with diabetes.

Whatever you choose to write, it is critical that you really focus on your **positive thoughts and feelings**. Ideally, we would like you to focus on feelings, thoughts, or changes that you have not discussed in great detail with others. You might also tie your thoughts and feelings about your experiences with diabetes to other parts of your life---your childhood, people you love, who you are, or who you want to be. Again, the most important part of your writing is that you really focus on your **positive** emotions and thoughts.

Try to write without stopping for **15 minutes**. If you run out of things to say, just repeat what you have already written until the 15 minutes are up. Don't worry about grammar, spelling or sentence structure. Just write.
