# Supplementary material for: Web-Based Benefit-Finding Writing for Adults with Type 1 or Type 2 Diabetes: Preliminary Randomized Controlled Trial
Source: JMIR Diabetes. 2019 Jun 27;4(2):e13857. doi: 10.2196/13857 (PMC6620889; doi:10.2196/13857)
Supplement: Multimedia Appendix 3 [file diabetes_v4i2e13857_app3.pdf]

Multimedia Appendix 3. Results of secondary outcome measures: observed and estimated means, standard deviations at baseline, one-month and three-month follow-ups.

Outcome	Group	Observed Means (SD)			Estimated Means [d] (SD)		
Secondary Outcome		Baseline[a]	IMFU[b]	3MFU[c]	Baseline	IMFU	3MFU
**PHQ-9 (Depression)**							
	BFW	2.25 (1.62)	4.57 (4.50)	3.18 (2.81)	1.77 (0.49)	2.19 (0.83)	1.96 (0.73)
	CW	2.04 (1.58)	3.53 (3.08)	3.17 (3.04)	1.68 (0.48)	1.95 (0.90)	1.96 (0.90)
**GAD-7 (Anxiety)**							
	BFW	1.71 (1.73)	3.76 (3.75)	3.41 (2.76)	1.57 (0.49)	2.03 (0.73)	2.03 (0.73)
	CW	1.54 (1.71)	2.79 (2.33)	2.47 (1.97)	1.51 (0.48)	1.84 (0.83)	1.85 (0.83)
**Summary of Diabetes Self-Care Activities Measure (Revised) subscales**							
General Diet							
	BFW	4.93 (2.14)	5.00 (1.82)	4.94 (1.43)	6.10 (0.64)	6.01 (0.64)	5.95 (0.68)
	CW	5.46 (1.41)	5.29 (1.46)	5.48 (1.34)	6.22 (0.62)	6.15 (0.62)	6.21 (0.76)
Specific Diet (Fruit and Vegetable)							
	BFW	4.30 (2.34)	4.86 (2.06)	4.59 (1.91)	5.93 (0.73)	6.02 (0.68)	5.97 (0.73)
	CW	5.58 (1.87)	5.41 (1.69)	5.17 (1.80)	6.36 (0.69)	6.30 (0.69)	6.23 (0.83)
Specific Diet (High Fat Foods)							
	BFW	3.61 (2.45)	3.57 (1.90)	2.94 (2.19)	3.39 (2.20)	3.58 (2.20)	3.11 (2.35)
	CW	3.29 (2.03)	3.88 (2.17)	3.57 (1.93)	3.71 (2.15)	3.61 (2.37)	3.54 (2.70)
Exercise							
	BFW	3.39 (2.28)	2.76 (2.18)	3.76 (2.26)	3.39 (2.30)	2.85 (2.15)	3.67 (2.40)
	CW	3.98 (2.25)	3.85 (2.07)	4.15 (2.20)	3.98 (2.29)	3.67 (2.22)	4.18 (2.56)
Blood Glucose Testing							
	BFW	5.50 (2.27)	5.33 (2.22)	6.00 (2.16)	6.61 (0.49)	6.57 (0.49)	6.74 (0.49)
	CW	5.56 (2.31)	5.72 (2.23)	5.20 (2.83)	6.64 (0.48)	6.67 (0.48)	6.65 (0.55)

[a] BFW n = 24, CW n = 48; [b]BFW n = 21, CW n = 33; [c]BFW n = 17, CW n = 23; [d]Estimated means based on ITT sample of BFW n=24, CW n =48, with variables transformed (log or square root) to address skewness, Standard deviations and confidence intervals are shown in parentheses.

Multimedia Appendix 3 continued. Results of secondary outcome measures: observed and estimated means, standard deviations at baseline, one-month and three-month follow-ups.

Outcome	Group	Observed Means (SD)			Estimated Means [d] (SD)		
Secondary Outcome		Baseline[a]	IMFU[b]	3MFU[c]	Baseline	IMFU	3MFU
**Summary of Diabetes Self-Care Activities Measure (Revised) subscale**							
Foot Care							
	BFW	2.17 (2.67)	1.83 (2.10)	2.26 (2.47)	2.17 (2.59)	1.93 (2.49)	1.96 (0.73)
	CW	2.70 (2.45)	3.51 (2.51)	2.89 (2.52)	2.70 (2.49)	3.36 (2.56)	1.96 (0.90)
**Self-Reported Health**							
	BFW	4.00 (0.80)	3.86 (0.91)	3.94 (0.83)	4.00 (0.83)	3.86 (0.88)	3.93 (0.88)
	CW	3.94 (0.84)	4.03 (0.87)	3.87 (0.81)	3.94 (0.83)	4.10 (0.90)	3.91 (0.97)
**Health Care Utilization**							
	BFW	2.04 (1.97)	2.86 (3.35)	2.47 (2.74)	0.92 (0.68)	1.02 (0.78)	0.94 (0.73)
	CW	1.71 (2.28)	1.82 (2.30)	1.61 (1.34)	0.76 (0.62)	0.73 (0.83)	0.81 (0.83)

[a] BFW n = 24, CW n = 48; [b]BFW n = 21, CW n = 33; [c]BFW n = 17, CW n = 23; [d]Estimated means based on ITT sample of BFW n=24, CW n =48, with variables transformed (log or square root) to address skewness, Standard deviations and confidence intervals are shown in parentheses.
