# Supplementary material for: Web-Based Benefit-Finding Writing for Adults with Type 1 or Type 2 Diabetes: Preliminary Randomized Controlled Trial
Source: JMIR Diabetes. 2019 Jun 27;4(2):e13857. doi: 10.2196/13857 (PMC6620889; doi:10.2196/13857)
Supplement: Multimedia Appendix 4 [file diabetes_v4i2e13857_app4.pdf]

Multimedia Appendix 4. Estimated differences in mean change of secondary outcomes between baseline and one-month follow-up, and between baseline and three-month follow-up, for the benefit-finding writing (BFW) group versus the control writing (CW) group.

Outcome	C1: Baseline to one-month follow-up			C2: Baseline to three-month follow-up		
	Estimated Mean Difference (95% CI)	*P*	Cohen's *d* effect size *d* (95% CI)	Estimated Mean Difference (95% CI)	*P*	Cohen's *d* effect size *d* (95% CI)
**PHQ-9 (Depression)**						
	- 0.18 (-0.57 to 0.22)	.38	0.31 (-0.24 to 0.74)	0.06 (-0.37 to 0.48)	.79	- 0.19 (-0.67 to 0.31)
**GAD-7 (Anxiety)**						
	- 0.14 (-0.51 to 0.23)	.46	0.27 (-0.23 to 0.76)	- 0.12 (-0.99 to 0.24)	.50	0.25 (-0.25 to 0.74)
**rSDSCA subscales**						
General Diet						
	0.02 (-0.23 to 0.27)	.89	- 0.03 (-0.52 to 0.46)	0.15 (-0.13 to 0.43)	.30	- 0.22 (-0.70 to 0.29)
Specific Diet (Fruit and Veg)						
	- 0.14 (-0.39 to 0.10)	.24	0.21 (-0.28 to 0.71)	- 0.17 (-0.57 to 0.22)	.38	0.24 (-0.27 to 0.72)
Specific Diet (High Fat Foods)						
	-0.28 (-1.26 to 0.70)	.57	0.13 (-0.36 to 0.62)	0.12 (-1.11 to 1.34)	.85	- 0.05 (-0.54 to -0.44)
Exercise						
	0.23 (-0.36 to 0.82)	.44	- 0.10 (-0.59 to 0.39)	- 0.08 (-0.99 to 0.83)	.86	0.04 (-0.53 to 0.45)
Blood Glucose Testing						
	0.08 (-0.08 to 0.23)	.34	- 0.14 (-0.64 to 0.35)	- 0.12 (-0.33 to 0.09)	.26	0.25 (-0.25 to 0.74)
Foot Care						
	0.91 (-0.00 to 1.83)	.61	- 0.36 (-0.85 to 0.14)	0.27 (-0.77 to 1.30)	.61	-0.10 (-0.59 to 0.39)
**Self-Reported Health**						
	0.31 (0.01 to 0.60)	.044	- 0.36 (-0.84 to 0.14)	0.05 (-0.35 to 0.44)	.81	-0.05 (-0.53 to 0.45)
**Health Care Utilization**						
	- 0.13 (-0.42 to 0.15)	.36	0.20 (-0.30 to 0.68)	0.03 (-0.40 to 0.46)	.90	- 0.05 (-0.53 to 0.45)

rSDSCA Revised Summary of Diabetes Self Care Activities

C1: Planned contrast 1: Difference between BFW and CW groups in change from baseline to 1-month follow-up: BFW (Baseline – 1MFU) – CW (Baseline – 1MFU)
C2: Planned contrast 2: Difference between BFW and CW groups in change from baseline to 3-month follow-up BFW (Baseline – 3MFU) – CW (Baseline – 3MFU)
