# Supplementary material for: Web-Based Benefit-Finding Writing for Adults with Type 1 or Type 2 Diabetes: Preliminary Randomized Controlled Trial
Source: JMIR Diabetes. 2019 Jun 27;4(2):e13857. doi: 10.2196/13857 (PMC6620889; doi:10.2196/13857)
Supplement: Multimedia Appendix 5 [file diabetes_v4i2e13857_app5.pdf]

Multimedia Appendix 5. Within-group estimated changes in secondary outcomes between baseline and one-month follow-up, and between baseline and three-month follow-up.

Outcome	C3: Baseline to one-month follow-up			C4: Baseline to three-month follow-up		
Group	Estimated Mean Difference (95% CI)	*P*	Cohen's *d* effect size *d* (95% CI)	Estimated Mean Difference (95% CI)	*P*	Cohen's *d* effect size *d* (95% CI)
**PHQ-9 (Depression)**						
BFW Group	-0.45 (-0.76 to -0.13)	.006	0.62 (0.04 to 1.20)	-0.22 (-0.55 to 0.11)	.19	0.31 (-0.26 to 0.86)
CW Group	-0.27 (-0.51 to -0.02)	.03	0.38 (-0.03 to 0.78)	-0.28 (-0.54 to 0.01)	.04	0.43 (0.03 to 0.83)
**GAD-7 (Anxiety)**						
BFW Group	-0.46 (-0.75 to -0.19)	.003	0.74 (0.12 to 1.33)	-0.46 (-0.75 to -0.17)	.002	0.74 (0.12 to 1.33)
CW Group	-0.32 (-0.55 to -0.09)	.006	0.49 (0.08 to 0.89)	-0.34 (-0.75 to -0.17)	.006	0.50 (0.10 to 0.91)
**rSDSCA subscales**						
General Diet						
BFW Group	0.09 (-0.10 to 0.29)	.34	-0.14 (-0.71 to 0.43)	0.15 (-0.06 to 0.37)	.13	-0.23 (-0.80 to 0.34)
CW Group	0.07 (-0.08 to 0.22)	.35	-0.11 (-0.51 to 0.29)	0.01 (-0.17 to 0.18)	.94	-0.01 (-0.42 to 0.39)
Specific Diet (Fruit and Veg)						
BFW Group	-0.08 (-0.28 to 0.10)	.39	0.13 (-0.44 to 0.69)	-0.04 (-0.35 to 0.27)	.79	0.06 (-0.51 to 0.62)
CW Group	0.06 (-0.09 to 0.21)	.41	-0.09 (-0.49 to 0.31)	0.13 (-0.11 to 0.38)	.29	-0.17 (-0.57 to 0.23)
Specific Diet (High Fat Foods)						
BFW Group	-0.19 (-0.96 to 0.59)	.63	0.09 (-0.48 to 0.65)	0.29 (-0.66 to 1.20)	.55	-0.12 (-0.69 to 0.44)
CW Group	0.10 (-0.50 to 0.69)	.75	-0.04 (-0.44 to 0.36)	0.17 (-0.60 to 0.94)	.66	-0.07 (-0.47 to 0.33)
Exercise						
BFW Group	0.54 (0.07 to 1.01)	.02	-0.24 (-0.81 to 0.33)	-0.28 (-0.10 to 0.42)	.43	0.12 (-0.45 to 0.69)
CW Group	0.31 (-0.05 to 0.67)	.09	-0.14 (-0.54 to 0.26)	-0.20 (-0.79 to 0.39)	.50	0.08 (-0.32 to 0.48)

BFW: Benefit-Finding Writing; CW: Control Writing; rSDSCA Revised Summary of Diabetes Self Care Activities
C3: Planned contrast 3: Within-group changes from baseline to 1-month follow-up: Baseline – 1-month follow-up
C4: Planned contrast 4: Within-group changes from baseline to 3-month follow-up: Baseline – 3-month follow-up

Multimedia Appendix 5 continued. Within-group estimated changes in secondary outcomes between baseline and one-month follow-up, and between baseline and three-month follow-up.

Outcome	C3: Baseline to one-month follow-up			C4: Baseline to three-month follow-up		
Group	Estimated Mean Difference (95% CI)	*P*	Cohen's *d* effect size *d* (95% CI)	Estimated Mean Difference (95% CI)	*P*	Cohen's *d* effect size *d* (95% CI)
**rSDSCA subscales continued**						
Blood Glucose Testing						
BFW Group	0.04 (-0.08 to 0.17)	.48	-0.08 (-0.65 to 0.48)	-0.13 (-2.59 to 0.13)	.12	0.27 (-0.30 to 0.83)
CW Group	-0.03 (-0.13 to 0.07)	.52	0.06 (-0.34 to 0.46)	-0.01 (-0.14 to 0.13)	.91	0.02 (-0.38 to 0.42)
Foot Care						
BFW Group	0.25 (-0.48 to 0.98)	.50	-0.09 (-0.66 to 0.47)	-0.02 (-0.80 to 0.80)	0.10	0.00 (-0.56 to 0.57)
CW Group	-0.66 (-1.22 to -0.11)	.02	0.26 (-0.14 to 0.66)	-0.27 (-0.92 to 0.38)	.41	0.10 (-0.30 to 0.50)
**Self-Reported Health**						
BFW Group	0.14 (-0.09 to 0.38)	.23	-0.16 (-0.73 to 0.40)	0.07 (-0.23 to 0.38)	.64	-0.08 (-0.65 to 0.48)
CW Group	-0.16 (-0.35 to 0.02)	.08	0.19 (-0.22 to 0.59)	-0.02 (-0.22 to 0.27)	.85	-0.03 (-0.43 to 0.37)
**Health Care Utilization**						
BFW Group	-0.10 (-0.33 to 0.12)	0.367 NS	0.14 (-0.43 to 0.70)	-0.02 (-0.36 to 0.32)	0.904 NS	0.03 (-0.54 to 0.59)
CW Group	0.03 (-0.15 to 0.21)	0.734 NS	-0.04 (-0.44 to 0.36)	-0.04 (-0.32 to 0.22)	0.719 NS	0.07 (-0.33 to 0.47)

BFW: Benefit-Finding Writing; CW: Control Writing; rSDSCA Revised Summary of Diabetes Self Care Activities

C3: Planned contrast 3: Within-group changes from baseline to 1-month follow-up: Baseline – 1-month follow-up

C4: Planned contrast 4: Within-group changes from baseline to 3-month follow-up: Baseline – 3-month follow-up
